# Implementation of evidence-based practice for alcohol and substance use disorders: protocol for systematic review

**DOI:** 10.1186/s13643-020-1285-0

**Published:** 2020-02-07

**Authors:** Eva Louie, Emma L. Barrett, Andrew Baillie, Paul Haber, Kirsten C. Morley

**Affiliations:** 1 Faculty of Medicine and Health, Discipline of Addiction Medicine, Central Clinical School, Sydney Medical School, The University of Sydney, Sydney, NSW 2006 Australia; 2 The Matilda Centre for Research in Mental Health and Substance Use, Faculty of Medicine and Health, The University of Sydney, Sydney, NSW Australia; 3 Faculty of Health Sciences, The University of Sydney, Sydney, NSW Australia; 4 Drug Health Services, Royal Prince Alfred Hospital, Sydney, NSW Australia

**Keywords:** Implementation, Alcohol, Substance use, Addiction, Systematic review

## Abstract

**Background:**

Whilst effective treatments exist for substance use and alcohol use disorders, they are not commonly practised. Studies have shown that only a small percentage of services provide evidence-based treatments such as addiction medications or psychosocial therapies. Although there is a growing body of literature on evidence-based treatment, no synthesis of research on the implementation of evidence-based addiction treatment exists. This proposed systematic review will synthesise and evaluate the effectiveness of implementation programmes in the treatment of patients with drug and alcohol problems using the Consolidated Framework for Implementation Research (CFIR) framework.

**Methods:**

We will search (from inception onwards) PubMed/MEDLINE, Cochrane Library, PsycINFO, Web of Science and CINAHL. Eligible studies will be clinical trials (e.g. randomised controlled trials, non-randomised controlled trials) and observational studies (e.g. before-and-after studies, interrupted time series) evaluating strategies used to implement evidence-based psychosocial treatments for alcohol and substance use disorders. The primary outcomes will be related to the implementation, service system, or clinical practice (e.g. acceptability, implementation costs, feasibility). Two researchers will independently screen all citations, full-text articles and abstract data. Risk of bias of individual studies will be appraised using appropriate tools. A narrative synthesis will be provided.

**Discussion:**

This project aims to provide evidence to help guide the design of translational research programmes to improve implementation of evidence-based care in drug and alcohol settings. Findings from the study will specify effective strategies for domains of influence including (1) intervention characteristics (e.g. evidence strength and quality, adaptability), (2) outer setting (e.g. patient needs and resources, external policies and incentives), (3) inner setting (e.g. implementation climate, readiness for implementation), (4) individuals involved (e.g. self-efficacy, knowledge and beliefs about the intervention) and (5) the implementation process (e.g. engaging members of the organisation, executing the innovation). Identified gaps in knowledge will guide further study.

**Systematic review registration:**

PROSPERO CRD42019123812

## Background

The existence of a gap between clinical evidence and clinical practice is well established in the health services literature [1, 2]. Studies from the USA, the Netherlands, Britain, Canada and Australia suggest that 30 to 40% of patients do not receive evidence-based treatments, and up to 25% of patients receive treatments that are either inappropriate or even harmful [3, 4]. The general trends in the gap between research and practice are equally evident in the treatment of addiction where evidence-based treatments are not widely practised [5–7]. Specifically, it has been estimated that no more than 25% of community-based services provide evidence-based treatments such as addiction medications, psychosocial therapies, or integrated services for clients with substance use disorders (SUDs) or co-occurring mental health disorders [8]. Whilst effective treatments exist for SUDs, they are not commonly practised [7, 9, 10].

A complex range of barriers operating at multiple levels of healthcare delivery must be addressed in order to bridge this gap, such as the patient level, the provider team or group level, the organisational level and the market or policy level [11]. Historically, there has been a much stronger focus on identifying evidence-based *interventions* than there has been on developing evidence-based *implementation* strategies [12]. Interventions that are validated under controlled conditions may not be effective when implemented in substance use disorder practice settings, whereby some features impact the acceptability, uptake and appropriateness of innovations and demand a level of flexibility in the approach that may compromise the efficacy of the intervention [13, 14]. For an intervention to be successful, it must therefore be feasible within the context it is intended so that the fidelity of the intervention can be reproduced and maintained [15].

The field of implementation science emerged accordingly as an organised and resourced approach to the study of implementation, allowing for the accumulation of knowledge about how to ensure that evidence-based practices are delivered to the necessary patients and thus bridging the gap between evidence and practice [12]. Without implementation science and the knowledge that it provides about the effectiveness of the implementation process, it is not possible to tell whether poor treatment outcomes are due to the intervention or the way it was implemented [16, 17]. The addictions field is grossly underrepresented within implementation science [8], despite high disease burden [18] and large gap between treatment and evidence. Thus, there is a particular need to apply this science to the implementation of evidence-based treatment of SUDs.

Various theories, models and frameworks have been proposed to synthesise the knowledge base derived from implementation research, to describe the process of translating research into practice, to understand the determinants of implementation outcomes and to evaluate implementation [19]. The Consolidated Framework for Implementation Research [17] has been recommended as a taxonomy that may be appropriate for use in the context of SUD research [20]. The Consolidated Framework for Implementation Research (CFIR) consolidates the plethora of terms and concepts generated by implementation researchers into five domains of influence: (1) intervention characteristics (e.g. evidence strength and quality, adaptability), (2) outer setting (e.g. patient needs and resources, external policies and incentives), (3) inner setting (e.g. implementation climate, readiness for implementation), (4) individuals involved (e.g. self-efficacy, knowledge and beliefs about the intervention) and (5) the implementation process (e.g. engaging members of the organisation, executing the innovation). It has been suggested that the CFIR could assist with differentiating the core components from the adaptive components of the intervention [7, 21], utilising formative evaluation in implementation research and developing and testing models that can assist with the prediction of implementation outcomes and sustainability in specific contexts [17]. Additionally, the CFIR has been used to assess the comprehensiveness of strategies employed to implement evidence-based practice in health and mental health settings [22, 23]. When compared to other theoretical approaches, it has been categorised as a ‘determinant framework’ because it is comprised of domains of determinants that are related to implementation outcomes [19]. Amongst the determinant frameworks included in this category, the CFIR provides the most comprehensive approach to synthesising implementation research. The CFIR has therefore been selected as the evaluation framework for this review given its appropriateness, as well as its utility in previous reviews and the SUD context in particular.

The number of empirical evaluations testing implementation strategies in mental health settings is ‘dwarfed’ by the broader field of health care [22]. This argument is particularly pertinent in the SUD setting, where implementation research is lacking [24]. Existing reviews have tended to focus on prevention (e.g. [25, 26]), treatment efficacy (e.g. [27, 28]) and specific interventions (e.g. [29]). Those that have specifically identified implementation strategies have addressed specific factors (e.g. [30]) or relationships between factors (e.g. [31]) influencing the implementation but have not provided a comprehensive account of implementation effectiveness. We now aim to synthesise information on implementation strategies utilised in the SUD field in general and using the CFIR framework to guide analysis.

The objectives of this systematic review are to synthesise and evaluate the effectiveness of implementation programmes in the treatment of patients with drug and alcohol problems.

We aim to evaluate implementation effectiveness with regards to the five domains of influence outlined by the CFIR framework. By providing this overview, we aim to provide evidence to help guide the design of translational research programmes to improve implementation of evidence-based care in drug and alcohol settings.

## Methods

The present review protocol is being reported in accordance with the reporting guidance the Preferred Reporting Items for Systematic Reviews and Meta-Analyses Protocols (PRISMA-P) statement [32] (see PRISMA-P checklist in Additional file 1). This review protocol was registered within the International Prospective Register of Systematic Reviews (PROSPERO) (registration number CRD42019123812).

### Eligibility criteria

Criteria for considering studies for this review are classified by the following:

#### Population

In order to meet inclusion criteria, studies must involve an evaluation of implementation strategies used to transfer an evidence-based psychosocial treatment or treatment guideline into clinical practice in SUD settings. Psychosocial treatments include any attempt to affect change in patients’ substance use through behaviour, cognition, affect, interpersonal relationships or environment (e.g. employment, housing). Participants in these studies may include any clinician providing psychosocial interventions to SUD patients accessing outpatient or inpatient drug and alcohol services. ‘Clinician’ is defined as an individual employed to implement change in SUD patients’ substance use using psychosocial treatments exclusively. As such, studies will be excluded from the review if they focus on the development of psychometric instruments, drugs in sport, harm prevention or community awareness.

#### Intervention

The psychosocial intervention must be evidence-based and provide clear recommendations for practice. Studies will be excluded if they involve physiological, pharmacological or education-based interventions. Information including the nature of desired change, strategies employed, source of the intervention, mode of delivery (individual or group), identification of who delivered the intervention and the timing, duration, and frequency of the intervention needs to be stated clearly. Only ethically approved studies will be considered.

#### Comparator and study design

Only studies with a comparison group will be included. Comparisons may be made before and after the administration of the intervention, between two or more forms of intervention, or between different types of intervention(s) (or no intervention). We will include randomised controlled trials (RCTs), non-randomised controlled trials, observational studies including before-and-after studies, and time series analyses.

#### Outcomes

Primary study outcomes will be related to the implementation, service system or clinical practice. Specifically, outcomes will include infidelity, attitudes toward or satisfaction with the intervention (acceptability), and adoption, appropriateness of the intervention to the target population, implementation costs, the feasibility of the intervention within the setting, and the sustainability of the intervention after implementation. The length of post-intervention follow-up period must be specified and any possible ceiling effects identified. Outcomes will be related to the effectiveness of the implementation process, as distinct from the efficacy of the intervention itself.

#### Setting

Since SUD inpatient and outpatient treatment settings that provide counselling services to patients are the focus of the review, settings such as primary care, criminal justice or those investigating cross-cultural factors will be excluded from the review.

### Information sources

The following electronic databases will be searched (from inception onwards): PubMed/MEDLINE, Cochrane Library, PsycINFO, Web of Science and CINAHL. Additionally, we will conduct reference searches of relevant reviews and articles. Similarly, a grey literature search will be done with help of Google and the Grey Matters tool which is a checklist of health-related sites organised by topic. The tool is produced by the Canadian Agency for Drugs and Technologies in Health (CADTH) [33].

### Search strategy

The search will include all relevant peer-reviewed studies. The search will be conducted across 4 relevant concepts (see draft strategy in Additional file 2): (1) implementation, (2) evidence-based practices, (3) substance use service setting/drug and alcohol service setting and (4) eligible research designs. Specific terms used to search these concepts will be adapted from a recent systematic review conducted in the mental health field [22]. The MEDLINE draft search strategy is available in Additional file 2.

### Selection and data extraction

Two reviewers will screen all articles identified from the search independently. First, titles and abstracts of articles returned from initial searches will be screened based on the eligibility criteria outlined above. Second, full texts will be examined in detail and screened for eligibility. Third, references of all considered articles will be hand-searched to identify any relevant report missed in the search strategy by two reviewers independently. Any disagreement between reviewers will be resolved by discussion to meet a consensus. EndNote version X9 (Clarivate Analytics) will be used to manage all records.

Two researchers will extract data and organise it into variables based on the Cochrane Effective Practice and Organisation of Care Review Group (EPOC) Data Abstraction Form (e.g. clinical interventions, strategies, outcomes and results), the conceptual model of Proctor et al. [12] (implementation, service system and clinical outcomes), information about any specific implementation frameworks used, and a checklist of items aligned with the domains and subdomains of the CFIR (i.e. subdomains associated with *intervention characteristics, outer setting, inner setting, characteristics of individuals* and the implementation *process*). This method was used effectively in two previous reviews [23, 34] as a means of categorising the types of implementation strategies addressed by each of the studies included in the review.

### Risk of bias of individual studies

All included studies will be critically evaluated by two researchers independently using the Revised Cochrane risk-of-bias tool (RoB 2) [24]. The RoB 2 provides a systematic assessment across five domains of bias (the randomisation process, deviations from intended interventions, missing outcome data, measurement of the outcome and selection of the reported results) to assess quality of the article per outcome. For cluster-randomised studies, an additional domain must be used when assessing the randomization process. Any non-randomised studies of interventions will be assessed using the ROBINS-I tool is a risk of bias tool [35].

### Data synthesis

We do not anticipate having sufficient studies for a meta-analysis and therefore plan to perform a narrative synthesis. The main methods of synthesis will most likely involve tabulation, textual descriptions, qualitative synthesis of themes and vote-counting [36]. We will synthesise the findings from the included articles using the CFIR framework. If any issues requiring sensitivity analyses arise during the review process, such analyses will be conducted.

### Meta-bias

Meta-biases such as outcome reporting bias will be evaluated by ensuring that a protocol was published prior to the recruitment of participants. Trial registries will also be checked to determine the integrity of reported outcome measures and statistical methods. The grey literature search may also assist with identifying publication bias.

### Confidence in cumulative evidence

Strength of the evidence will be graded according to the Grading of Recommendations Assessment, Development and Evaluation (GRADE) approach [37]. In the event that there is disagreement between the two reviewers, a third researcher will be consulted.

## Discussion

This manuscript provides a detailed account of this systematic review protocol used to advance effective components of implementation programmes in treating addiction. To our knowledge, this is the first systematic review of implementation studies for drug and alcohol settings for a range of evidence-based approaches. There has been one previous review of implementation of SUD treatment [38] that specifically focused on one type of service provision, integrated care. We wish to look at implementation of evidence-based practice more broadly and to utilise an implementation science framework to synthesise studies. A review of this kind is an essential component of bridging the gap between clinical research and practice for drug and alcohol services. This review will provide a summary of the implementation research that has been conducted in SUD settings to date, provide recommendations and will identify areas that are lacking research evidence. This review will provide a foundation from which to develop innovative methods for implementing evidence-based practice in drug and alcohol healthcare settings.

Given the complex, multi-level nature of implementing evidence-based practice, the results of this review will inform several stakeholders. For instance, those making decisions at the economic or policy level, organisational leadership, provider group management and clinicians being required to implement changes will be interested in the most effective means of translating evidence into practice. Researchers seeking to differentiate the efficacy of treatment outcomes from implementation outcomes will also benefit from the findings of this review, and several future research questions will be identified.

Potential limitations at the study and review level include the diversity of implementation strategies, types of clinical interventions, study conditions and outcomes found in the included articles. This will create challenges for the reviewers with regard to accurately synthesising and comparing the information. This may become particularly evident as the authors are attempting to ascertain which components of a particular intervention are the most effective means of achieving certain desired outcomes, or in situations that call for an understanding of how a specific strategy might influence the effectiveness of the implementation. On the other hand, the use of the EPOC data form, the conceptual model of Proctor et al. [12] and the CFIR [17] may assist with teasing out these inconsistencies. In addition, it is also possible that many outcomes relevant to the implementation may be underutilised or absent in the studies captured. Any protocol amendments will be documented in a protocol addendum and in the final manuscript of the systematic review.

Despite the challenges that may be encountered, this review will make a valuable contribution to the growing body of literature on the implementation of evidence-based practice in SUD settings. Importantly, it will provide a summary of the most effective means of increasing the use of evidence-based treatments amongst drug and alcohol clinicians and subsequently assist with reducing the burden of disease associated with SUDs. This information will make a significant contribution to understanding how to improve the quality of treatment offered for SUD in specialised settings and in turn to improve the outcomes of people seeking treatment for these disorders.

## Supplementary information

**Additional file 1:.** PRISMA-P 2015 Checklist.

**Additional file 2:.** Draft search strategy.

## Data Availability

Not applicable

## Abbreviations

CFIR: Consolidated Framework for Implementation Research
EPOC: Cochrane Effective Practice and Organisation of Care Review Group
GRADE: Grade of Recommendations Assessment, Development and Evaluation approach
PRISMA-P: Preferred Reporting Items for Systematic Reviews and Meta-Analyses Protocols
RCT: Randomised controlled trial
SUD: Substance use disorder
