# Supplementary material for: Implementation of evidence-based practice for alcohol and substance use disorders: protocol for systematic review
Source: Syst Rev. 2020 Feb 7;9:25. doi: 10.1186/s13643-020-1285-0 (PMC7007686; doi:10.1186/s13643-020-1285-0)
Supplement: Supplementary file 1 — Additional file 1:. PRISMA-P 2015 Checklist. [file 13643_2020_1285_MOESM1_ESM.docx]

# **This checklist has been adapted for use with systematic review protocol submissions to BioMed Central journals from Table 3 in Moher D et al:** Preferred reporting items for systematic review and meta-analysis protocols (PRISMA-P) 2015 statement. Systematic Reviews 2015 **4**:1

| **Section and topic** | **Item No** | **Checklist item** |
| --- | --- | --- |
| **Administrative information** |  |  |
| Title:   - Identification - Update | 1a  1b | Identify the report as a protocol for a systematic review  **Page 1, lines 3-4**  If the protocol is for an update of a previous systematic review, identify as such. **Not applicable** |
| Registration: | 2 | If registered, provide the name of the registry (e.g. PROSPERO) and registration number in the abstract  **Page 2, line 59-61** |
| Authors:   - Contact - Contributions | 3a  3b | Provide name, institutional affiliation, e-mail address of all protocol authors; provide physical mailing address of corresponding author  **Page 1, lines 7-22 and appendix 1 lines 541-545**  Describe the contributions of the protocol authors and identify the guarantor of the review  **Page 9, lines 368-372** |
| Amendments | 4 | If the protocol represents an amendment of a previously completed or published protocol, identify as such and list changes; otherwise, state plan for documenting important protocol amendments  **Not applicable** |
| Support:   - Sources - Sponsor - Role of sponsor or funder | 5a  5b  5c | Indicate sources of financial or other support for the review  **Page 8, line351-352**  Provide name for the review funder and/or sponsor  **Not applicable**  Describe the role of funder(s), sponsor(s), and/or institution(s), if any, in developing the protocol  **Not applicable** |
| **Introduction** |  |  |
| Rationale | 6 | Describe the rationale for the review in the context of what is already known  **Page 3-4, lines 72-143** |
| Objectives | 7 | Provide an explicit statement of the question(s) the review will address with reference to participants, interventions, comparators, and outcomes (PICO)  **Page 4, lines 145-150** |
| **Methods** |  |  |
| Eligibility criteria | 8 | Specify the study characteristics (e.g., PICO, study design, setting, time frame) and report characteristics (e.g., years considered, language, publication status) to be used as criteria for eligibility for the review  **Page 5-6, lines 160-205** |
| Information sources | 9 | Describe all intended information sources (e.g., electronic databases, contact with study authors, trial registers, or other grey literature sources) with planned dates of coverage  **Page 6, lines 207-213** |
| Search Strategy | 10 | Present draft of search strategy to be used for at least one electronic database, including planned limits, such that it could be repeated  **Page 6, line 215-221** |
| Study records:   - Data management - Selection process - Data collection process | 11a  11b  11c | Describe the mechanism(s) that will be used to manage records and data throughout the review  **Page 6, lines 230**  State the process that will be used for selecting studies (e.g., two independent reviewers) through each phase of the review (i.e., screening, eligibility, and inclusion in meta-analysis)  **Page 6, lines 223-229**  Describe planned method of extracting data from reports (e.g., piloting forms, done independently, in duplicate), any processes for obtaining and confirming data from investigators  **Page 6, lines 231-239** |
| Data items | 12 | List and define all variables for which data will be sought (e.g., PICO items, funding sources), any pre-planned data assumptions and simplifications  **Page 6, lines 231-239** |
| Outcomes and prioritization | 13 | List and define all outcomes for which data will be sought, including prioritization of main and additional outcomes, with rationale  **Page 6, lines ???** |
| Risk of bias in individual studies | 14 | Describe anticipated methods for assessing risk of bias of individual studies, including whether this will be done at the outcome or study level, or both; state how this information will be used in data synthesis  **Page 7, lines 241-276** |
| Data |  |  |
| Synthesis | 15a  15b  15c  15d | Describe criteria under which study data will be quantitatively synthesized  **Not applicable**  If data are appropriate for quantitative synthesis, describe planned summary measures, methods of handling data, and methods of combining data from studies, including any planned exploration of consistency (e.g., *I* ^2^, Kendall’s tau)  **Not applicable**  Describe any proposed additional analyses (e.g., sensitivity or subgroup analyses, meta-regression)  **Not applicable**  If quantitative synthesis is not appropriate, describe the type of summary planned  **Page 7, lines 278-284** |
| Meta-bias(es) | 16 | Specify any planned assessment of meta-bias(es) (e.g., publication bias across studies, selective reporting within studies)  **Page 7, lines 286-290** |
| Confidence in cumulative evidence | 17 | Describe how the strength of the body of evidence will be assessed (e.g., GRADE)  **Page 7, lines 292-295** |
