# Supplementary material for: Implementation of evidence-based practice for alcohol and substance use disorders: protocol for systematic review
Source: Syst Rev. 2020 Feb 7;9:25. doi: 10.1186/s13643-020-1285-0 (PMC7007686; doi:10.1186/s13643-020-1285-0)
Supplement: Supplementary file 2 — Additional file 2:. Draft search strategy. [file 13643_2020_1285_MOESM2_ESM.docx]

**Table S1**

| **Concept** | **Description** | **Search Terms** |
| --- | --- | --- |
|  | Implementation | exp implementation/ OR exp adoption OR quality improvement.mp/ OR complex intervention.mp/ OR exp dissemination/ OR technology transfer.mp/ OR exp diffusion/ OR exp education/ |
|  | Evidence-based practices | empirically supported treatment.mp/ OR evidence based practice.mp/ OR evidence based treatment.mp/ OR evidence based intervention.mp/ OR best practice.mp/ OR exp innovation/ OR exp guideline/ OR exp training/ OR evidence-based medicine.mp/ |
|  | Substance Use service setting/Drug and Alcohol service setting | exp alcohol/ OR illicit drugs.mp/ OR exp cannabis/ OR exp heroin/ OR exp methamphetamine/ OR exp substance/ OR exp misuse/ OR abuse/ OR drug depend.mp/ OR exp addict/ OR alcohol and other drug.mp/ OR drug and alcohol.mp/ OR substance-related disorders.mp/ AND  exp treatment/ OR exp provider/ OR exp agency/ OR exp program/ OR exp rehabilitation/ OR exp detoxification/ OR exp withdrawal/ OR primary care.mp/ OR behavioural care.mp/ OR housing service.mp/ OR general practice.mp/ OR social care.mp/ |
|  | Eligible research designs | randomized controlled trial.mp/ OR randomised controlled trial.mp/ OR exp RCT OR controlled clinical trial.mp/ OR exp CCT/ OR controlled before and after study.mp/ OR exp CBA/ OR interrupted time series.mp OR exp ITS/ OR stepped-wedge trial.mp/ OR exp SWT/ |

(Search was limited to “Humans” and “English Language”)

- Search: 1+2+3+4
